# Supplementary figures and images for: Genome sizes and repeatome evolution in zoantharians (Cnidaria: Hexacorallia: Zoantharia)
Source: PeerJ. 2023 Oct 16;11:e16188. doi: 10.7717/peerj.16188 (PMC10586311; doi:10.7717/peerj.16188)

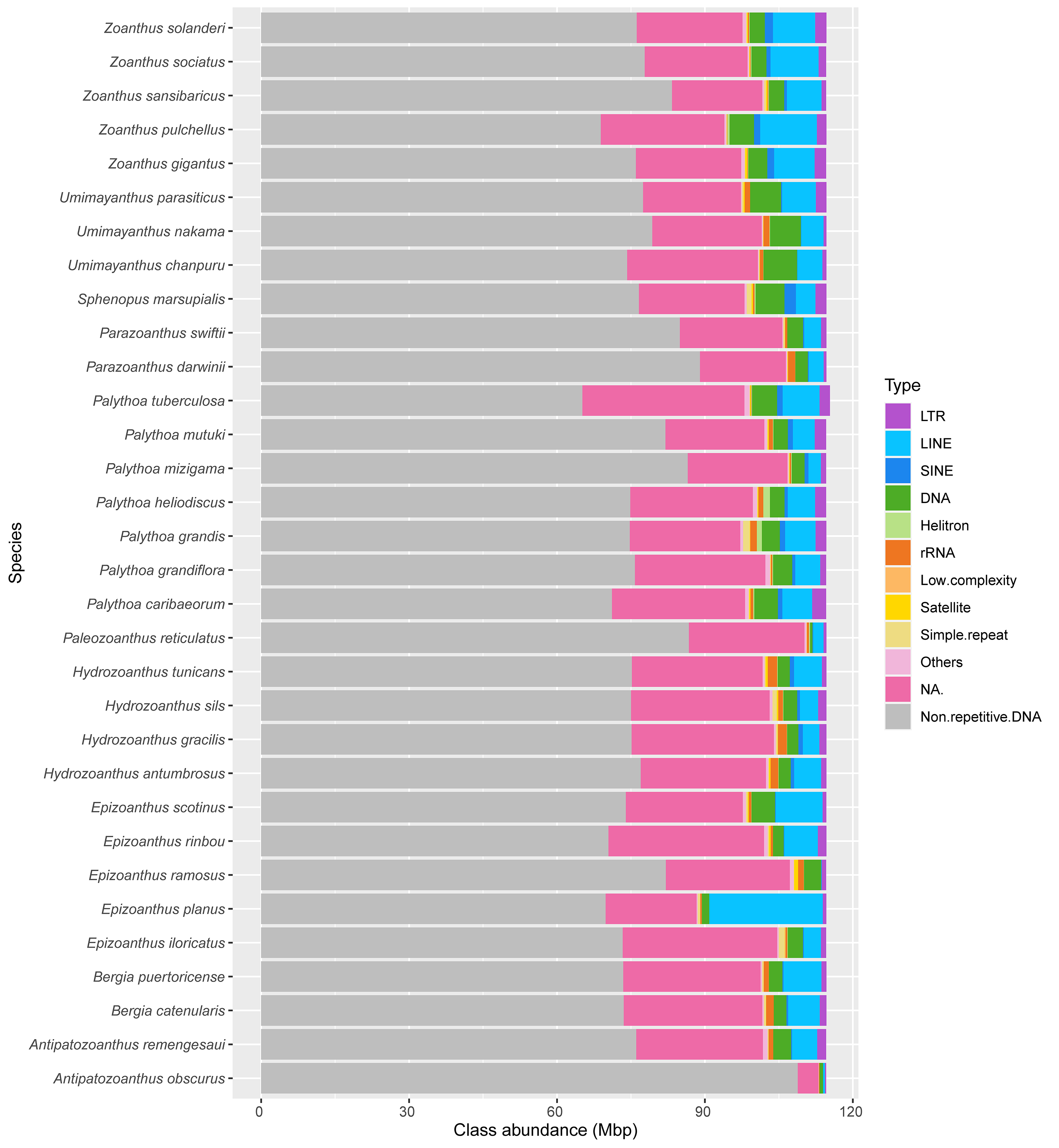

Supplement: Supplemental Information 1 — This includes the results of species for which genome size estimates could not be produced, and hence were not displayed in Fig. 1. [file peerj-11-16188-s001.png]

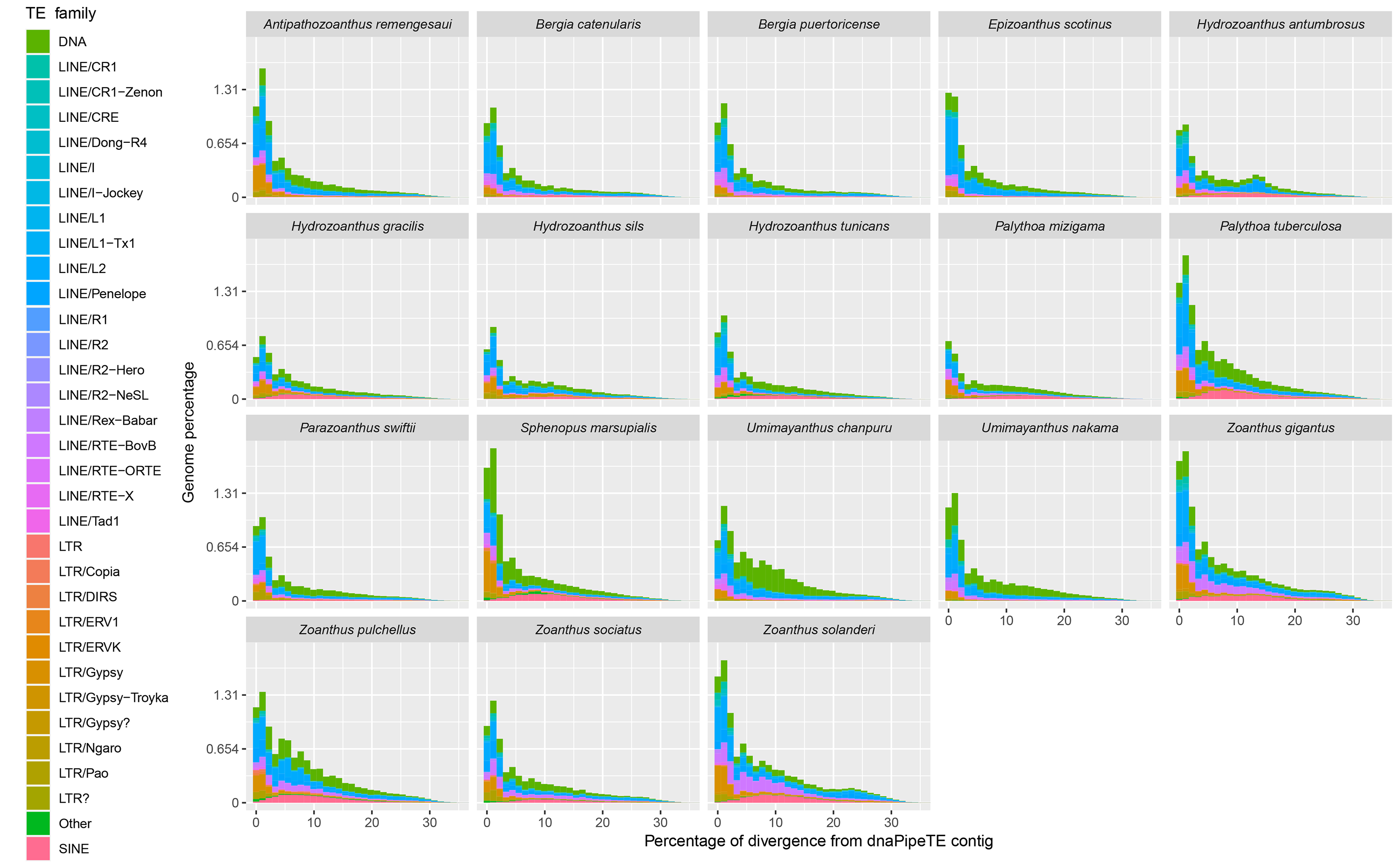

Supplement: Supplemental Information 2 [file peerj-11-16188-s002.png]

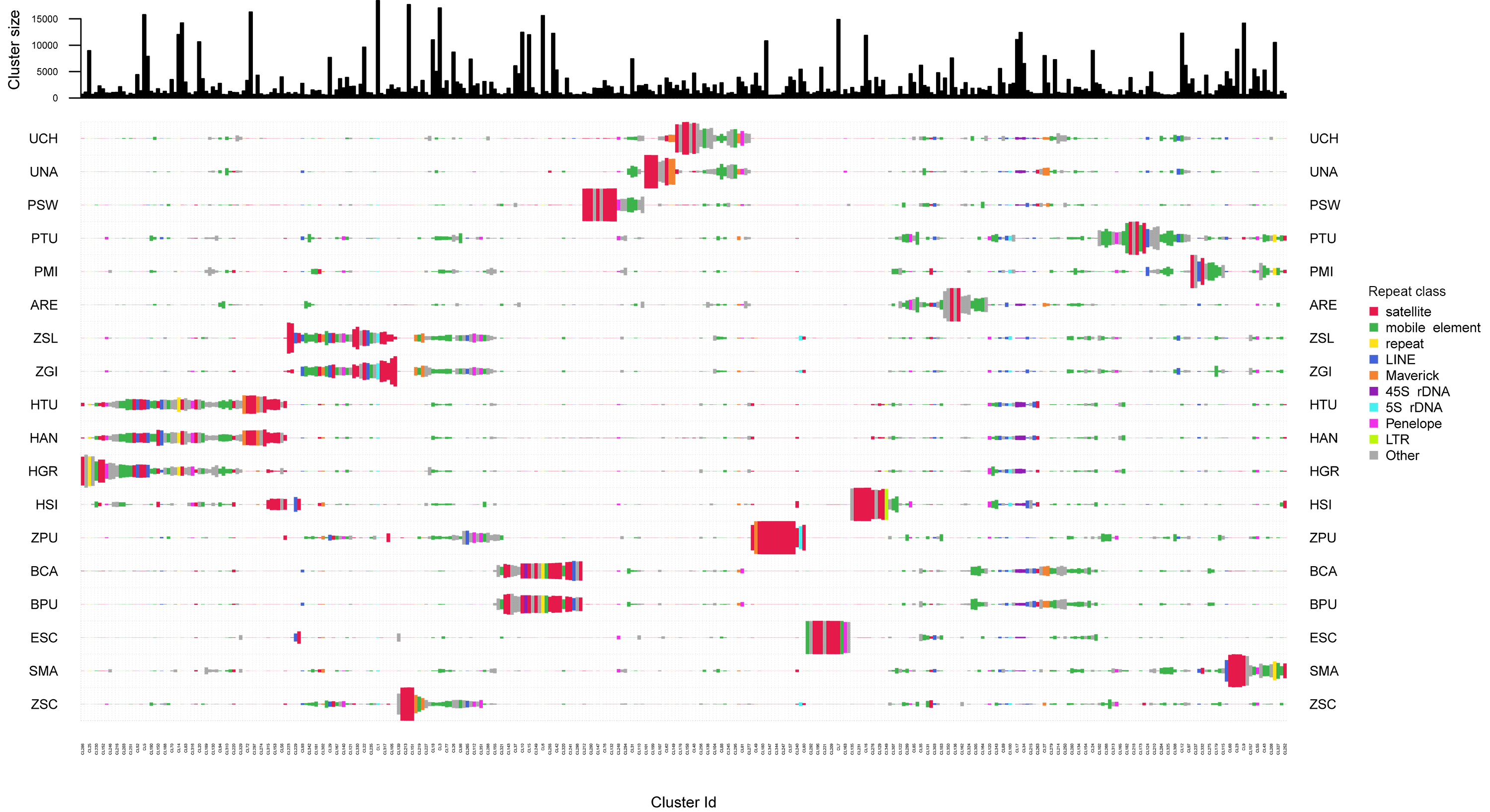

Supplement: Supplemental Information 3 — Species names are shown as three letter codes. U. chanpuru: UCH; U. nakama: UNA; P. swiftii: PSW; P. tuberculosa: PTU; P. mizigama: PMI; A. remengesaui: ARE; Z. solanderi: ZSL; Z. gigantus: ZGI; H.tunicans: HTU; H. antumbrosus: HAN; H. gracilis: HGR; H. sils: I; Z. pulchellus: ZPU; B. catenularis: BCA; B. puertoricense: BPU; E. scotinus: ESC; S. marsupialis: SMA; Z. sociatus: ZSC. [file peerj-11-16188-s003.png]

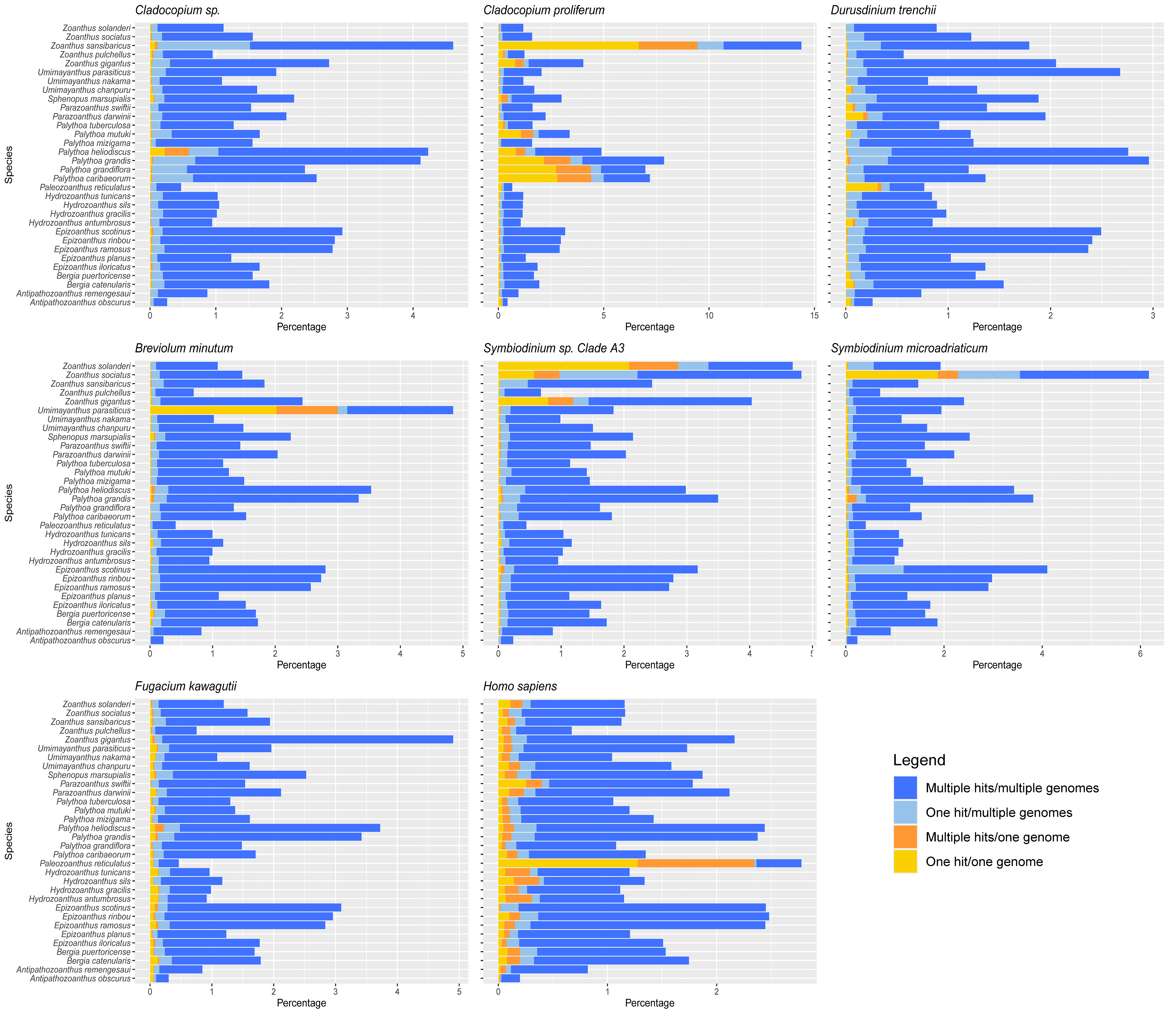

Supplement: Supplemental Information 4 — Each zoantharian dataset was simulataneously screened for contamination against 7 available Symbiodiniaceae genomes as well as human genome. The screening involved mapping of 100,000 zoantharian reads onto potential contaminant genomes, which are here reported in percentage of reads mapping. The number of reads that mapped onto a single contaminant genome are coloured in yellow (one hit in the genome) and orange (several hits in same genome). The number of reads that mapped onto several different contaminant genomes are coloured in light (one hit) and dark blue (multiple hits). Reads mapping onto a single genome are to be considered as serious contamination whereas reads mapping onto several genomes at the same time can represent the presence of similar regions between target and potential contaminant genomes. [file peerj-11-16188-s004.png]
